# Generation of Human-Induced Pluripotent Stem Cells by a Nonintegrating RNA Sendai Virus Vector in Feeder-Free or Xeno-Free Conditions

**DOI:** 10.1155/2012/564612

**Published:** 2012-03-22

**Authors:** Chad C. MacArthur, Andrew Fontes, Namritha Ravinder, David Kuninger, Jasmeet Kaur, Matthew Bailey, Antje Taliana, Mohan C. Vemuri, Pauline T. Lieu

**Affiliations:** ^1^Primary and Stem Cell Systems, Life Technologies Corporation, 5781 Van Allen Way, Carlsbad, CA 92008, USA; ^2^Molecular and Cell Biology, Life Technologies Corporation, 5781 Van Allen Way, Carlsbad, CA 92008, USA; ^3^Primary and Stem Cell Systems, Life Technologies Corporation, 7335 Executive Way, Frederick, MD 21704, USA

## Abstract

The generation of induced pluripotent stem cells (iPSCs) from somatic cells has enabled the possibility of providing unprecedented access to patient-specific iPSC cells for drug screening, disease modeling, and cell therapy applications. However, a major obstacle to the use of iPSC for therapeutic applications is the potential of genomic modifications caused by insertion of viral transgenes in the cellular genome. A second concern is that reprogramming often requires the use of animal feeder layers and reagents that contain animal origin products, which hinder the generation of clinical-grade iPSCs. Here, we report the generation of iPSCs by an RNA Sendai virus vector that does not integrate into the cells genome, providing transgene-free iPSC line. In addition, reprogramming can be performed in feeder-free condition with StemPro hESC SFM medium and in xeno-free (XF) conditions. Generation of an integrant-free iPSCs generated in xeno-free media should facilitate the safe downstream applications of iPSC-based cell therapies.

## 1. Introduction

Takahashi and Yamanaka first demonstrated that induced pluripotent stem cells (iPSCs) can be generated from somatic cells by transducing four transcription factors using retroviral vectors [1, 2]. This breakthrough in creating induced pluripotent stem cells opened the door for personalized medicine using patient-derived iPSCs [3–6]. The major limitation for potential clinical application is the integration of viral transgenes into the host genome that can result in multiple insertions and risk of tumorigenicity [7, 8]. Another major disadvantage of reprogramming cells with integrating vectors is that silencing and activation of transgenes are unpredictable, which may affect terminal differentiation potential and increase the risk of using iPSC-derived cells. Multiple methods have been developed to address these issues, including reprogramming with episomal vectors, mRNAs, miRNAs, protein transduction, or treatment with chemical compounds [9]. The majority of these methods has one or more limitations, such as low reprogramming efficiency or requiring multiple rounds of transfections, or is effective only with specific cell types, such as skin fibroblasts. We have previously shown that sustained expression of reprogramming factors is required for at least 10–20 days [10], and often these reprogramming methods fail to sustain expression and are difficult to repeat. Further, due to poor efficiency of existing methods, reprogramming often has been performed in the presence of animal feeders (inactivated mouse embryonic fibroblasts) to maximize colony formation [11] along with the use of serum and xeno-containing products, which are not ideal for clinical applications [11, 12].

 Here, we investigate the use of Sendai virus vector to generate transgene-free iPSCs in different conditions. Sendai virus vector is a negative-strand RNA virus that belongs to the *paramyxoviridae *family [13–15]. Unlike other RNA viruses, it replicates in the cytoplasm of infected cells and does not go through a DNA phase that can integrate into the host genome [13]. In addition, Sendai virus vector can infect a broad host range and is nonpathogenic to humans. Sendai virus vector has been considered for clinical studies of gene therapy for cystic fibrosis [16, 17] and vaccine delivery [18]. Recent papers also demonstrated that Sendai virus vector can reprogram somatic cells with higher efficiency than other reprogramming methods [19]. Thus, the nature of Sendai virus vector makes it an ideal tool for cell reprogramming and stem cell research.

The current methods of reprogramming often require the use of inactivated feeders and animal-derived products during several steps to maximize reprogramming efficiency. However, exposure of human cells to animal origin products may increase the risk of nonhuman pathogen transmission and immune rejection [20], hence limiting their use in clinical settings. Given the reported high efficiency of Sendai virus vector in generating pluripotent cells, we investigated the generation of transgene-free iPSCs with Sendai virus vector in feeder-free conditions using StemPro hESC SFM medium and in xeno-free conditions. Several reports have demonstrated the generation of iPSCs in animal product-free culture media but their performance tends to be lower than knockout serum (KO-SR) based media [21]. Here, we report a robust and an efficient system to generate transgene-free iPSCs in different conditions, and its ease of use can be applied to a wide range of different cell types. Generation of transgene-free iPSCs under these conditions will be important to facilitate the safe clinical translation of iPSC-based therapies.

## 2. Materials and Methods

### 2.1. Cell Culture

Human neonatal fibroblasts (ATCC Cat. no. CRL-2252) were maintained in fibroblast growth media consisting of DMEM + GlutaMAX (Cat. no. 10569-010), supplemented with 10% ES cell-qualified fetal bovine serum (Cat. no. 16141-079), and 1% nonessential amino acids (Cat. no. 11140-050). Feeder-free iPSC cultures were maintained on hESC-qualified GelTrex (Cat. no. A10480-02) with chemically defined StemPro hESC SFM (Cat. no. A1000701) supplemented with 100 *μ*M 2-mercaptoethanol (Cat. no. 21985-023) and 10 ng/mL recombinant human basic FGF (Cat. no. PHG0264). For xeno-free culture, human neonatal fibroblasts were maintained in StemPro MSC SFM Xeno-Free medium (Cat. no. A10675-01). Xeno-free iPSC cultures were maintained on KnockOut-DMEM/F-12 (Cat. no. 12660012) supplemented with 15% KnockOut-SR Xeno-Free CTS (Cat. no. A1099202), 1X KnockOut SR Xeno-Free Growth Factor Cocktail, 2 mM GlutaMAX (Cat. no. 35050-061), 1% nonessential amino acids, 100 *μ*M 2-mercaptoethanol, and 20 ng/mL of bFGF. For feeder condition, human iPS cells were maintained on irradiated mouse embryonic fibroblasts (Cat. no. S1520-100) in DMEM/F-12 + GlutaMAX (Cat. no. 10565-018) supplemented with 20% KnockOut SR (Cat. no. 10828-028), 1% nonessential amino acids, 100 *μ*M 2-mercaptoethanol, and 4 ng/mL of bFGF. iPSCs were passaged manually using a 26-gauge needle or StemPro EZPassage Disposable Stem Cell Passaging Tool (Cat. no. 23181-010). All reagents are from Life Technologies. Colonies were passaged every 3-4 days at a splitting ratio of 1 : 3. The Gibco Episomal hiPSC line was purchased from Life Technologies (Cat. no. A13777) and maintained on irradiated mouse embryonic fibroblasts in DMEM/F-12 + GlutaMAX supplemented with 20% KnockOut SR, 1% nonessential amino acids, 100 *μ*M 2-mercaptoethanol, and 4 ng/mL bFGF.

### 2.2. Generation of iPSCs in Feeder-Free and Xeno-Free Conditions

For the generation of iPSCs in feeder-free condition, approximately 100,000 human neonatal fibroblasts were seeded per well in a 6-well plate and incubated at 37°C and 5% CO_2_. Two days later, cells were transduced with the CytoTune iPS Reprogramming Kit containing Sendai virus vectors using the F gene-defective vector as described in [19, 22] (Life Technologies Cat. no. A1378001) in fibroblast growth medium at an MOI of 3, as described in the manufacturer's protocols. One day after transduction, the medium was replaced with fresh fibroblast growth medium and the cells were cultured in fibroblast growth media for 6-7 days. On day eight after transduction, cells were transferred to 10 cm Geltrex-coated tissue culture dishes at 5 × 10^5^ cells per dish. After culturing overnight in fibroblast growth media, the medium was replaced daily with StemPro hESC SFM medium. The iPSC colonies were picked for expansion and characterization from days 17–21 of reprogramming.

For the generation of iPSCs in xeno-free condition, approximately 500,000 human neonatal fibroblasts were seeded in StemPro MSC SFM Xeno-Free medium per well in a 6-well plate and incubated at 37°C and 5% CO_2_. The next day, cells were transduced with the CytoTune iPS Reprogramming Kit in StemPro MSC SFM Xeno-free medium at an MOI of 3. One day after transduction, the medium was replaced with fresh StemPro MSC SFM Xeno-Free medium and cells were cultured for 6-7 days. On day eight after transduction, cells were transferred to 10 cm tissue culture dishes plated with inactivated human feeders (NuFF, GlobalStem Cat. no. GSC-3001G) at a density of 2 × 10^5^ cells per dish. After culturing overnight, the medium was replaced daily with xeno-free iPSC medium. The xeno-free medium is composed of KnockOut D-MEM CTS containing 15% KnockOut SR Xeno-Free CTS, 2 mM GlutaMAX, KnockOut SR GF Cocktail CTS (used at 1 : 50), and 8 ng/mL bFGF. Just prior to use, medium was equilibrated at 37°C and 5% CO_2_ in humidified air, 100 *μ*M 2-mercaptoethanol was added to the KnockOut SR Xeno-Free Complete Medium. The iPSC colonies were picked for expansion and characterization from days 21–25 of reprogramming.

### 2.3. Immunofluorescence and Alkaline Phosphatase Staining

For immunofluorescence staining, cells were fixed in 2% PFA for 30 minutes at room temperature, blocked for 30 minutes at room temperature in blocking buffer (5% normal goat serum, 1% BSA, and 0.1% Triton X100 in D-PBS), incubated with primary antibodies (diluted in blocking buffer) overnight at 4°C, washed 3X with D-PBS, incubated with secondary antibodies (diluted in D-PBS) for 30 minutes at room temperature, and washed 3X with D-PBS. For live staining, cells were washed 1X with DMEM/F-12 and incubated with primary antibody at 1 : 100 dilution (in DMEM/F-12) for 60 min at 37°C. Cells were washed 3X with DMEM/F12 and incubated with secondary antibody at 1 : 500 dilution (in DMEM/F-12) for 60 minutes at 37°C and washed 3X with DMEM/F-12. Characterization was carried out using the following antibodies: Mouse anti-Tra1-60 antibody (Cat. no. 41–1000), Mouse anti-Tra1-81 antibody (Cat. no. 41–1100), Mouse anti-SSEA4 (Cat. no. 41–4000), Alexa Fluor 488 goat anti-mouse IgG (H + L) antibody (Cat. no. A11029), Alexa Fluor 594 goat anti-mouse IgG (H + L) antibody (Cat. no. A11032), Alexa Fluor 488 goat anti-rabbit IgG (H + L) antibody (Cat no. A11034), and Alexa Fluor 594 goat anti-rabbit IgG (H + L) antibody (Cat no. A11037). All reagents are from Life Technologies. Clones were screened for residual virus using rabbit anti-SeV antibody (MBL International Corporation, Woburn, MA; Cat. no PD029). Images were captured using a Zeiss Axiovision microscope and processed using AdobePhotoshop CS. Where applicable, on day 25 of reprogramming, cells were stained using VECTOR Red Alkaline Phosphatase Substrate Kit (Vector Labs Cat. no. SK 5100) and positive colonies were counted.

### 2.4. RNA Isolation, qRT-PCR, and End Point PCR for Gene Expression Analysis

Total RNA was extracted from cells using TRIzol LS reagent (Cat. no. 10296-010) with DNaseI (Cat. no. AM2222). RT-PCR reactions were carried out using 20x Reverse Transcriptase enzyme mix and 2x RT Buffer provided in the TaqMan Gene Expression Cells-to-CT Kit (Cat. no. 4399002) with 1 ug of total RNA per reaction. Gene expression assays were performed according to the manufacturer's protocols. Primers for gene expression can be found at http://www.invitrogen.com/site/us/en/home.html. All reactions were performed in triplicates and normalized to endogenous B-actin. End point PCR was performed with 10 uL of cDNA in 50 uL of AccuPrime Pfx Supermix (Cat. no. 12344040) with the following primers to detect for the presence of Sendai virus vector (SeV: Frw: GGATCACTAGGTGATATCGAGC, Rev: ACCAGACAAGAGTTTAAGAGATATGTATC) as described in [19]. The expected size of the PCR product is 181 bp. All reagents are from Life Technologies. PCR was performed with the following conditions: denaturation 95°C, 30 sec, annealing: 55°C, 30 sec, elongation: 72°C, 30 sec, for 30–35 cycles. PCR products were analyzed on 2% agarose gel electrophoresis.

### 2.5. MicroRNA Profiling

Total RNA was extracted from cells using TRIzol LS reagent (Cat. no. 10296-010) with DNASE 1 (Cat. no. AM2222). 500 ng of total RNA was subjected to cDNA synthesis using TaqMan MicroRNA Reverse Transcription Kit (Cat. no. 4366597) and TaqMan Human MicroRNA Array Card A (Cat. no. 4398965) to quantify microRNA expression levels, according to the manufacturer's protocol. All reagents are from Life Technologies. Real-time PCR was performed on an AB 7900HT Sequence Detection System with cycling conditions of 95°C for 10 min followed by 95°C for 15 sec and 60°C for 60 sec for a total of 40 cycles. The Ct values for all miRNAs were normalized to mammalian U6 snRNA levels by calculating their respective delta Ct values. Relative changes in miRNA expression levels between samples were compared using the delta Ct values.

### 2.6. TaqMan Protein Assays and Reagents

All TaqMan Protein Assays and associated reagents kits were obtained from Life Technologies. Cells were harvested, counted, and 50 K cells were incubated in 100 uL of resuspension and 100 uL of lysis buffer. TaqMan Protein Assays were carried out with cell lysate dilutions of 500 or 250 cells per reaction using Applied Biosystems StepOnePlus real-time PCR system. In parallel, the same cell inputs were also subjected to 18S genomic ribosomal DNA assays to obtain endogenous control Cq, described in [10].

### 2.7. *In Vitro* Differentiation of iPSCs

 Undifferentiated iPSCs were harvested using collagenase to generate embryoid bodies (EBs) and were cultured for 4 days in suspension in differentiation medium containing DMEM-F12 with GlutaMAX, 20% Knockout Serum Replacement, 1% nonessential amino acid, and 55 *μ*M 2-mercaptoethanol (Life Technologies). On day 5, EBs were seeded on Geltrex coated plates for an additional 17 days of differentiation in the same medium, then the cells were used for immunocytochemistry. Induction to neural lineages was performed as described in [23].

## 3. Results and Discussion

### 3.1. Generation of iPSCs with Sendai Virus Vector in Feeder-Free or Xeno-Free Condition

**Figure 1:**
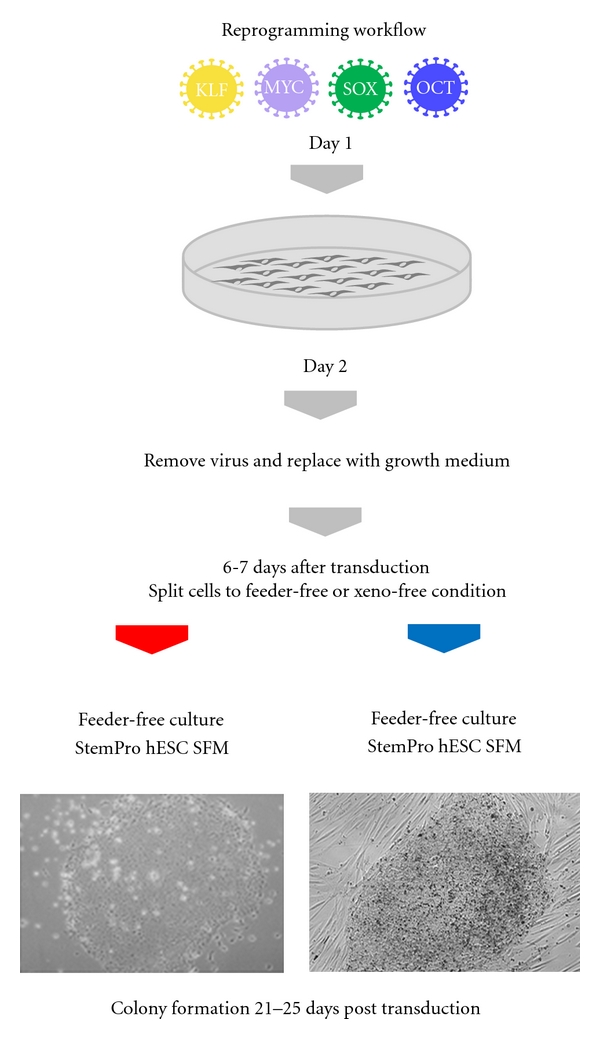
Reprogramming workflow on feeder-free and xeno-free conditions. (1) Transduce cells with CytoTune Sendai virus vector overnight. (2) Remove virus and replace with cell's growth medium. Cells are grown in cell's growth medium for 6-7 days after transduction. (3) Split cells onto Geltrex-coated plates or inactivated human feeders. Culture transduced cells in feeder-free conditions with StemPro hESC SFM medium and Geltrex-coated plates or xeno-free medium consisting of KnockOut-DMEM/F-12, 15% KnockOut-SR Xeno-Free CTS, and 1X KnockOut SR Xeno-Free Growth Factor Cocktail. Colonies can be picked between 17–25 days after transduction.

Generation of human iPSCs with Sendai virus vector encoding OCT3/4, KLF4, SOX2, and cMYC was performed on feeder-free conditions with StemPro hESC SFM and Geltrex coated plates, or xeno-free conditions on KnockOut-DMEM/F-12 supplemented with 15% KnockOut-SR Xeno-Free CTS, and 1X KnockOut SR GF Cocktail CTS on inactivated human feeders. Human neonatal foreskin fibroblast cells were plated onto a 6-well plate two days before transduction, and approximately 5 × 10^5^ cells were transduced with OCT3/4, KLF4, SOX2, and cMYC Sendai virus vector at an MOI of 3. About 6-7 days after transduction, cells were collected and transferred to Geltrex-coated matrix plate (feeder-free condition) or onto inactivated human feeders (xeno-free conditions) at the density of 500,000 cells per 10 cm dish for feeder-free condition and 200,000 cells per 10 cm dish for xeno-free conditions. The workflow is outlined in Figure 1, and viable colonies were visible as early as day 10 after transduction, and these colonies could be readily picked by 21–25 days.

**Figure 2:**
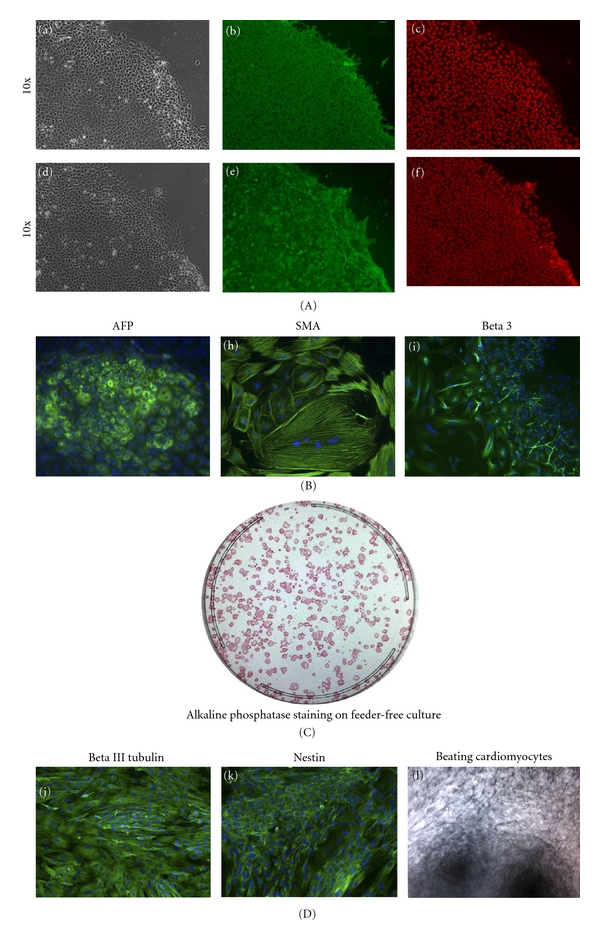
(A) Characterization and differentiation of iPSC colonies generated on feeder-free conditions. Colonies were stained for pluripotent markers: SSEA4 (b), SOX2 (c), Tra1-81 (e), NANOG (f), and phase ((a) and (d)). (B) These colonies were differentiated *in vitro* into embryoid bodies (EBs) comprising of the three embryonic germ layers: endoderm marker *α* fetoprotein (AFP) (g), mesodermal marker smooth muscle actin (SMA) (h), and ectoderm marker Beta 3-tubulin (Beta III) (i). (C) Colonies were stained with alkaline phosphatase staining 25 days after transduction. (D) Colonies were also subjected to directed differentiation to neural stem cells, stained with Beta 3-tubulin (Beta III) (j), Nestin (k), and random differentiation to beating cardiomyocytes (l).

**Figure 3:**
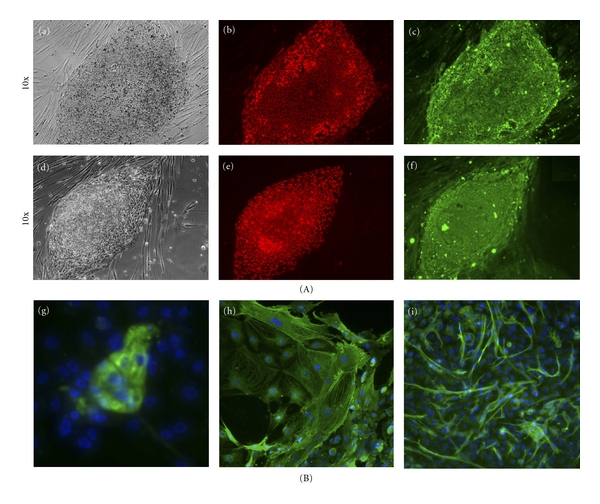
(A) Characterization and differentiation of iPSC colonies generated on xeno-free conditions. Colonies were stained for pluripotent markers: NANOG (b), SSEA4 (c), SOX2 (e), Tra1-60 (f), and phase ((a) and (d)). (B) These colonies were differentiated *in vitro* into embryoid bodies (EBs) comprising of the three embryonic germ layers: endoderm marker *α* fetoprotein (AFP) (g), mesodermal marker smooth muscle actin (SMA) (h), and ectoderm marker Beta 3-tubulin (Beta III) (i).

 A majority of colonies generated with Sendai virus vector have ES-like morphology, and very few transformed colonies were observed. Clones from human fibroblasts were stained with pluripotent markers, SSEA4, Sox2, Nanog, and Tra1-81 or Tra1-60 shown on Figure 2(A) (feeder-free condition) and Figure 3(A) (xeno-free condition). Alkaline phosphatase staining demonstrated efficiency on feeder-free condition to be between 0.01 and 0.04%, and very few transformed or non-iPSC colonies were detected, shown on Figure 2(C). In addition, these cells were able to give rise to embryoid bodies (EBs) that could differentiate to all the three lineages: ectoderm, endoderm, and mesoderm (see Figure 2(B) for an example in feeder-free conditions and Figure 3(B) for xeno-free conditions). We further performed directed induction of clones from feeder-free condition to neural lineages using N2B27 supplements as described in [23] and stained cells with beta III tubulin Figure 2(D) (j) and nestin (k), or by spontaneous differentiation that gave rise to beating cardiomyocytes, Figure 2(D) (l). The differentiation profile was similar to that seen with cells reprogrammed with other methods, and little variation between different subclones was seen.

### 3.2. Expression of Pluripotency Markers in Different iPSC Lines by TaqMan Gene Expression and Protein Assays

**Figure 4:**
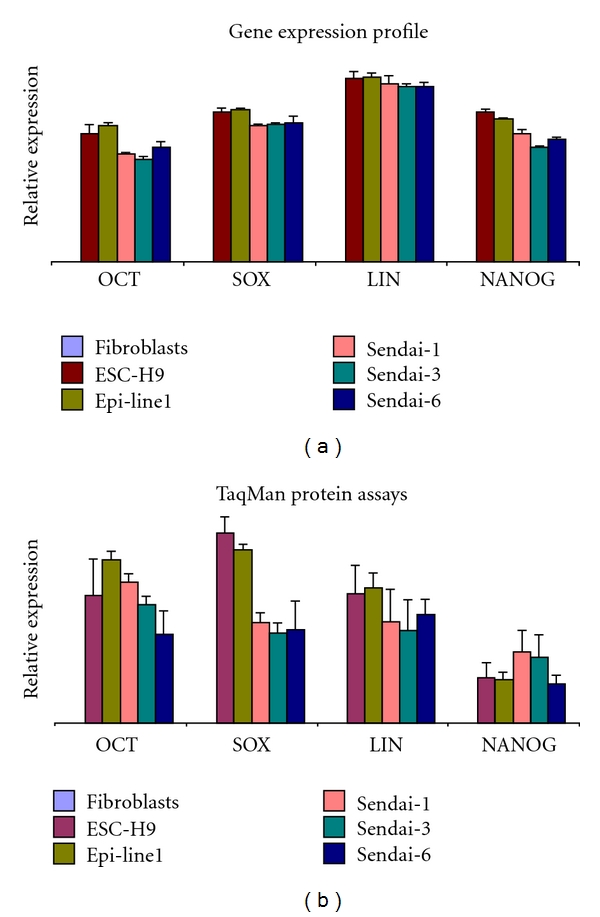
(a) TaqMan Gene Expression Assays of OCT4/POU5F1, SOX2, LIN28, and NANOG were compared among the WA09 hESC line, an episomally derived iPSC line (Epi-line 1), and three Sendai-derived iPSC lines (Sendai 1, 3, and 6). Samples were normalized against the endogenous reference B-actin. (b) TaqMan Protein Assays of OCT4/POU5F1, SOX2, LIN28, and NANOG are compared among the WA09 hESC line, an episomally derived iPSC line (Epi-line 1), and three Sendai-derived iPSC lines (Sendai 1, 3, and 6). Samples were normalized against the endogenous reference 18S ribosomal genomic DNA and plotted relative fold change to the expression level of untransduced human dermal fibroblasts.

We compared gene expression levels of OCT3/4, SOX2, NANOG, and LIN28 of iPSC lines generated by Sendai virus vector or by an episomal vector and found that expression profile was similar in all these lines as well as comparable to the WA09 embryonic stem cell line, shown on Figure 4(a). We previously demonstrated that TaqMan Protein Assays can be used to characterize different iPSC lines for the expression of pluripotent protein markers and have reported variations of protein levels among iPSC lines [10]. When iPSC lines generated from Sendai virus vector were compared to an episomal generated iPSC line [24] or the WA09 hESC line, there were subtle differences in the levels of protein expression for OCT3/4, SOX2, NANOG, and LIN28 between different iPSC lines. Noticeably, SOX2 levels of the Sendai-generated iPSC lines were lower than the episomal-generated iPSC line and the WA09 hESC line (Figure 4(b)). It is likely that the difference is due to the fact that the Sendai-generated iPSC lines were at earlier passages (P5–P10) compared to the episomal iPSC (>P20) or the WA09 hESC line. The variations in the levels of the markers were within the range we have observed in iPS lines generated with other methods [10], and in no case was the variation sufficiently large that the cells were not pluripotent as assessed by our *in vitro* assays. We noticed that the difference becomes less noticeable when the iPSC lines were at higher passages (data not shown). Recent publication by Chung et al. reports that human-induced pluripotent stem cells derived under feeder-free conditions display similar levels of gene expression as in hESCs [25]. In addition, the variability among iPSC lines is minimized when using a feeder-free culture system compared to on feeders [25].

### 3.3. MicroRNA Profiling

**Figure 5:**
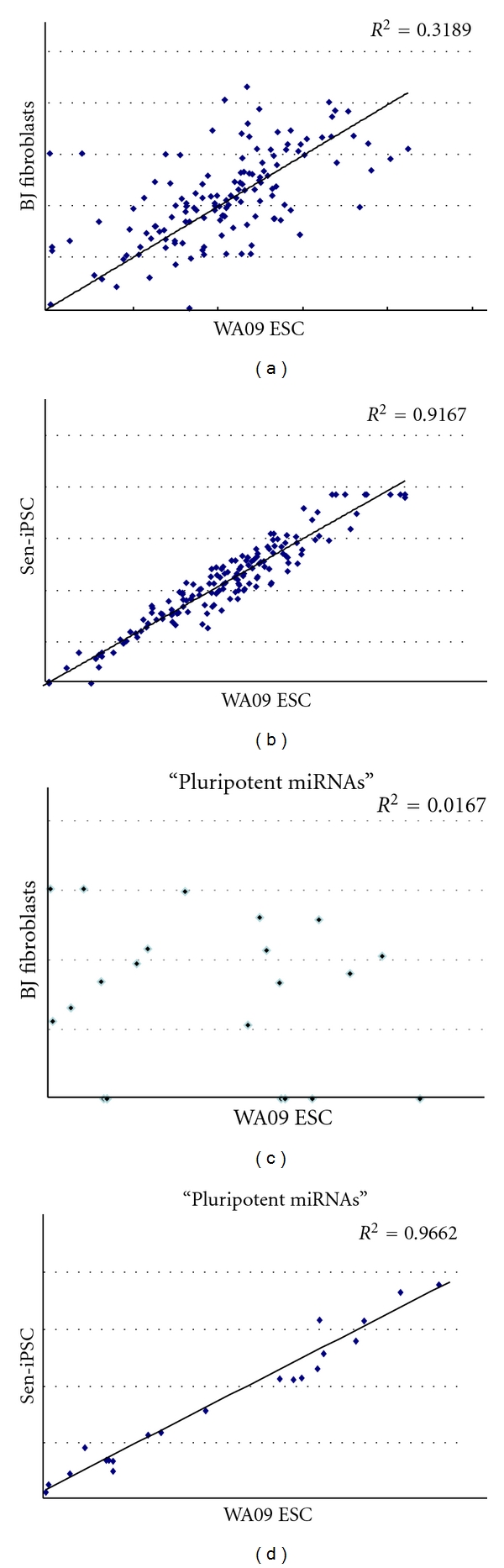
((a) and (b)) Scatter plots comparing microRNA expression profiles using the TaqMan Human MicroRNA Array of BJ fibroblasts, iPSCs generated with the Sendai virus vector (Sen-iPSC), and the WA09 human embryonic stem cell (hESC) line. ((c) and (d)) Scatter plots comparing “pluripotent” microRNA expression profiles.

 We performed miRNA profiling analysis with 384 unique miRNAs among three cell types, BJ fibroblasts, iPSCs generated with the Sendai virus vector, and the WA09 human embryonic stem cell (hESC) line. Data show poor correlation between BJ fibroblast and the WA09 human embryonic stem cell (hESC) line Figure 5 (*R* ^2^ = 0.3189). On the other hand, comparisons of the WA09 human embryonic stem cell (hESC) line and iPSC line generated from the Sendai virus vector were highly correlated though subtle differences in miRNA expression (*R* ^2^ = 0.9167), Figure 5, were observed. We also analyzed the “pluripotent” miRNAs identified previously [26, 27] in human iPSCs and hESCs, specifically miR-302, miR-367–371, mi-17–92, miR-299, and mi-Let 7 cluster, and found high correlation between the WA09 human embryonic stem cell (hESC) line and the iPSC line generated with Sendai virus vector (*R* ^2^ = 0.9662). These data demonstrate that the iPSC line generated with Sendai virus vector is very similar to the WA09 human embryonic stem cell (hESC) line as assessed by miRNA profiling by qPCR.

### 3.4. Detection of Virus Free iPSCs

**Figure 6:**
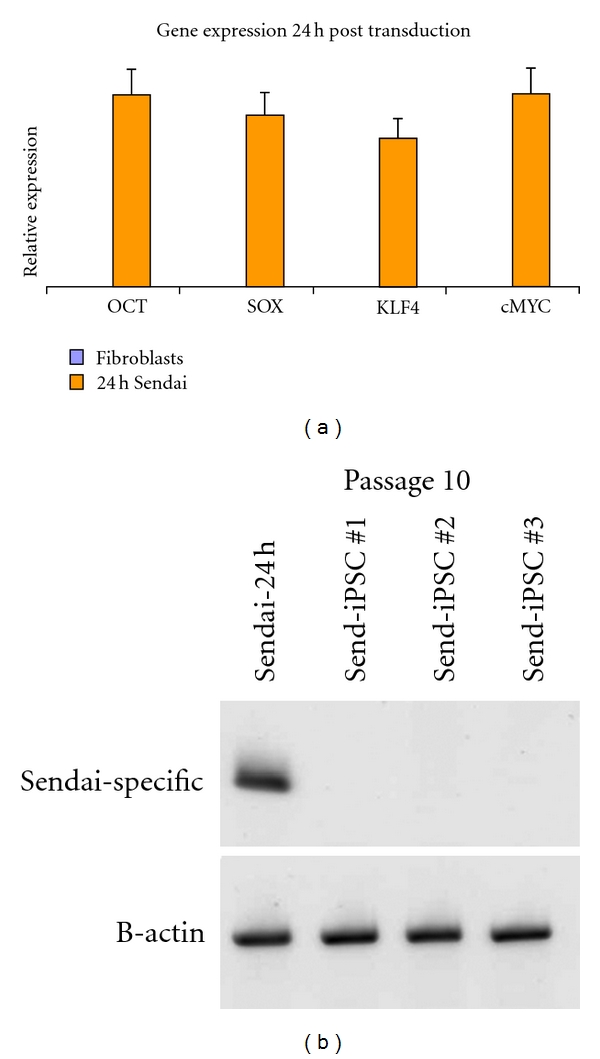
(a) Detection of viral gene expression at 24 hours after transduction with Sendai virus vector by TaqMan Gene Expression Assays. (b) Detection of virus-free iPSCs after 10 passages in culture by end-point PCR.

**Figure 7:**
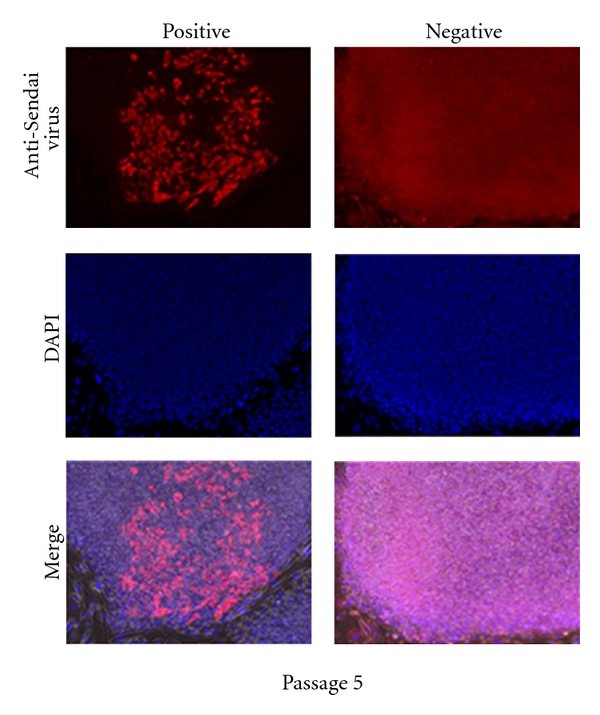
Immunofluorescence staining with anti-Sendai virus vector antibodies to detect virus-free iPSCs. iPSC clones were stained at passage 5.

 Since Sendai virus vector is an RNA virus that only replicates constitutively in the cytoplasm of infected cells [13], it carries no risk of modifying the host genome and it gradually lost as the cells proliferate. We used two different methods to detect loss of Sendai virus vector in cells. Using RT-PCR with Sendai specific primers, we can detect transgene-RNAs as early as 24 hours after transduction, Figure 6(a). The rate at which the transgenes disappeared varies between each clone, and transgene-free iPSC colonies were obtained by passage 10 using end point PCR method, Figure 6(b). The second method used to detect the presence of Sendai virus vector is by immunochemistry staining with anti-Sendai antibody. As early as passage 5, we can detect some colonies staining negative for Sendai virus vector, shown on Figure 7. Sendai virus vectors tend to dilute out and disappear as the clones are being carried out to further passages. By passage 10, residual Sendai transcripts were completely eliminated in three out of four clones tested. In addition, the Sendai backbone vector carries temperature-sensitive mutations that enable complete removal of the residual vector by shifting to nonpermissive temperature of 38-39°C [22]. Thus, removal of Sendai virus vector is less labor intensive compared to alternative methods that require subsequent excision of transgenes from the host genome [6, 28].

## 4. Conclusions

Sendai virus vector has been used as vectors for gene therapy since it has no risk of being integrated into the host genome and is nonpathogenic to humans. In addition, Sendai virus vector has been shown to generate transgene-free human iPSCs with high efficiency from fibroblasts and CD34+ cord blood cells [22]. In the present study, we demonstrated that the Sendai virus vector can be used to generate transgene-free human iPSCs in feeder-free and xeno-free conditions. Comparing the feeder-based and feeder-free systems, we noticed a decrease in reprogramming efficiency in feeder-free conditions, independent of which media we used. Since Sendai virus vector can reprogram with high efficiency, we are able to obtain sufficient number of colonies for further expansion in either condition. Another advantage of using Sendai virus vector is the ease of use with single transduction comparing to other methods that require multiple transfections [29]. Finally, iPSCs depleted of virus from the cytoplasm can be easily obtained without further manipulation. Thus, the high efficiency and ease of use of the Sendai virus vector is ideal for reprogramming with different cell types on feeder-based or feeder-free conditions.

Gene expression of pluripotent markers and miRNA profiling data show that iPSCs derived from Sendai virus vector on feeder-free condition are similar to hESC line (the WA09 embryonic stem cell line) and to another episomally derived iPSC line. Our data is consistent with the recent publication by Chung et al., which reports that feeder-free iPSCs have similar transcriptomic profiles and differentiation capacity to hESCs [25]. In addition, increasing evidence demonstrates the importance of mircoRNAs (miRNAs) for human embryonic stem cells self-renewal, pluripotency, and differentiation [27]. Previous publications reported a signature group of miRNAs that is upregulated in both iPSCs and hESCs, such as the miR-17–92 clusters, miR-290 clusters, miR-302, 367 cluster, miR-371/372/373, and let-7 family [26]. We further compared these “pluripotent” miRNAs and found high correlation between the human iPSCs generated by Sendai virus vector and the WA09 human embryonic stem cell (hESC) line. These data suggest that the nature of iPSCs generated with Sendai virus vector is similar to that of hESC line.

In summary, reports have shown that the use of integrating viruses to generate iPSCs is not ideal due to the potential of tumorigenicity and the exposure of iPSCs in animal containing products impedes downstream applications. It is important to develop a system that can generate iPSCs that are free of transgenes and in conditions that are free of animal-derived products. Exposure of human cells to animal origin products may increase immune rejection of grafted cells and make them unsuitable for the generation of clinical-grade iPSCs [11]. Here we have demonstrated that iPSCs can be generated in feeder-free or xeno-free conditions. Further, the iPSCs generated here are free of transgenes and behave similar to those generated on feeders or animal-derived products. The generation of a footprint-free iPSCs under these conditions should facilitate the safe clinical translation of iPSC-based therapies.
